# Evaluation of Serum Zinc Level Under Malignant Condition and its Possible Implication on Improving Cell-Mediated Immunity During Cancer Progression

**DOI:** 10.4021/wjon256e

**Published:** 2011-02-26

**Authors:** Prabir K. Chakravarty

**Affiliations:** Tumor Immunity and Gene therapy Unit, Chittaranjan National Cancer Institute, S.P. Mukherjee Road, Calcutta-700025, India

**Keywords:** Serum zinc, Cell-Mediated immunity, Cancer

## Abstract

**Background:**

Zinc is an essential trace element required for different biological functions in a living body. Evaluating its concentration and effect during malignancy is important not only to assess the disease activity but also to evaluate its role in some important biological activities like immunity.

**Methods:**

The experiments were carried out in transplanted, chemically induced and spontaneous tumors in mice. The serum zinc concentration (SZC) was determined under malignant condition and following treatment of solid tumor with anti-cancer agents. The importance of evaluating Cu/Zn ratio (CZR) was also determined. To evaluate its role in immune response during malignancy, studies were carried out on induced tumor bearing animals.

**Results:**

The results showed that SZC had an inverse correlation with the progression of the disease. It was significantly reduced in all forms of malignancy, more so in spontaneous and induced forms of the disease. Extensive studies done with the solid form of induced tumor, established the importance of estimating the concentration of Zn, in addition to that of copper, in assessing the disease activity following treatment with anti-cancer agents. With regression of the tumor mass, there was a significant elevation in the SZC and depression in the serum copper concentration (SCC) compared to their levels prior to treatment. This was also reflected on the value of copper zinc ratio (CZR); with the onset of tumor regression, a significant reduction in CZR was noted in the tumor bearing animals compared to their untreated levels. As the state of ‘regression’ in the tumor mass was maintained for a considerable period of time following treatment with anti-cancer agents along with elevated zinc levels, we considered the role of zinc in controlling the tumor growth, indirectly. We observed that zinc is able to stimulate lymphocyte proliferation that may directly impact on the immune response of tumor bearing host.

**Conclusions:**

Zinc appears to play a role in tumor progression and regression and in stimulating lymphocyte proliferation. It is hypothesized that supplementing zinc to malignant subjects prior to and following therapeutic intervention with anti-cancer agents could help improve immune response of the host against existing tumor.

## Introduction

Trace elements are known to play a pivotal role in the process of normal growth and differentiation of various tissues in animals and humans [1]. Their requirement for sustenance of tumor cell proliferation is hence considered to be of significant importance [2]. However, some of these elements do play a preventive role against malignant growth by involving in protection against oxidative stress which can generate free radicals in the cells that contribute to cancer development. Accumulation of free radicals has been implicated in the pathogenesis of many diseases including cancer [3]. Zinc is one such essential element that prevents the formation of free radicals. In addition to its role as an anti-oxidant, zinc is also known to participate in nearly 120 reactions taking place in a living organism [1]. More recently, studies are showing that this element may also play an important regulatory role in initiation of cell-mediated immunity [4-6]. Recent studies have demonstrated that Zn deficiency seriously inhibited the development of lymphoid organs, impaired the progression of lymphocytes from the G0/G1 phase to the S phase, and caused pathological injury in the lymphoid organs [7].

A consistent change in the level of different trace elements in the blood of patients with different forms of cancer has been noted by many investigators [8, 9]. This implied that variations in their concentration under malignant condition could also impact on tumor pathology, apart from their reported importance in diagnosis and prognosis of cancer. Among the trace elements, significant changes were observed for serum copper concentration (SCC) under various malignant conditions [10, 11]. As a result SCC is considered as a non-specific marker for monitoring the progression of malignant disease activity in some forms of tumor. We have reported in earlier studies on the usefulness of measuring SCC as an indicator of disease activity in different forms of cancer in human and in experimental tumors [10, 12]. However, the importance of determining zinc concentration during malignancy is not very well studied. Some earlier reports on the level of serum zinc concentration (SZC) during malignancy have been somewhat contradictory. While some have reported no change in the SZC [8], others have reported depressed values during malignancy [11]. To evaluate the role and importance of zinc during malignancy, we made an exhaustive study on the SZC in different forms of malignant conditions in animal models (transplanted, induced and spontaneous) and also examined the relevance of evaluating SZC and the copper/zinc ratio (CZR) in ascertaining the disease activity following successful treatment with anti-cancer agents. Further, we also explored the possible role of Zinc, if any, on lymphocyte proliferation which could impact on immune response of tumor bearing animals. The study was carried out in murine tumors of different origin. To study the effect of tumor regression on the trace element concentration, a solid tumor induced by a chemical carcinogen was used as a model tumor system. It is reported here that the SZC is consistently depressed in all forms of tumors studied, more so in the chemically induced form. The regression in the tumor volume following treatment with anti-cancer agents was reflected on the level of SZC and on copper/zinc ratio (CZR). The possible implications of Zn on the immune response have also been addressed.

## Materials and Methods

### Mice

Six to 8 week-old male strain A/RB and C_3_H female mice were obtained from the Institutes animal facility and maintained in the Animal Resources facilities of the Institute under controlled temperature, humidity, and a 12h light : dark cycle with food and water *ad libitum.*

### Tumor models

Studies were carried out with transplanted, chemically induced and spontaneous tumors. About 300 tumor bearing animals were used for the entire study.

(a) The transplanted tumors viz. sarcoma-180 (S-180), Earlich’s Ascites Carcinoma (EAC), and Dalton’s Lymphoma (DL), were maintained *in vivo* in the A/RB mice by serial passage. For the study animals received 1 x 10^7^ tumor cells or saline by ip injection in the abdomen. The tumor volume reached its maximum by day 17 and thereafter maintained a stagnant condition. The animals eventually died between 20 - 25 days following tumor transplantation. For our experiments, the animals were sacrificed on day 7, day 10 and day 17, when maximum tumor growth was noted. For evaluation of zinc in serum of control and tumor bearing animals about 150 animals were used. Forty-five animals were used in each transplanted group of tumors. Fifteen animals were used for assessing zinc concentration at different time points (7, 10 and 17 days following tumor cell transplantation respectively).

(b) The solid tumor was a fibro-sarcoma, induced by injecting 0.133% benz (a) pyrene suspension in olive oil into the right thigh of each mouse. Tumor growth was detected 3 to 4 months following injection, in 40% of the animals. Only the tumor bearing animals were included for the study. A portion of the tumor tissue was formalin fixed for H&E staining. The sections of the solid tumor were assessed for the tumor type. Only animals with fibro-sarcoma were used for the study.

(c) The spontaneous tumor was a mammary carcinoma in the female C_3_H mice. It is a spontaneously occurring tumor maintained in the animal facility of the Institute.

(d) Controls: Thirty age-matched male (strain A) and ten female (C_3_H) animals were used as control animals for the study.

### Chemotherapy

For chemotherapeutic study, forty animals (10 animals in each group) with chemically induced solid tumors were used. The tumor volume was measured prior to and following treatment with the two anti-cancer drugs: (a) 5-FU (Sigma Chemicals, St. Louise, MN) was given at a dose of 25 mg/kg body weight to the tumor-bearing animals for 7 consecutive days. (b) Mitomycin C (Sigma Chemicals, St. Louise, MN) was given at a dose of 8.4 mg/kg body weight. The entire dose was given in two separate injections at a week’s interval.

### Estimation of trace elements

For estimation of trace elements, after the specified period the animals were sacrificed by slitting the jugular vein. The serum was separated from the blood, and copper and zinc concentrations were determined by Atomic Absorption Spectrophotometry (AAS) as described previously [12]. Briefly, the serum was diluted 1:1 with deionised water and the trace element concentration was determined by AAS.

### Zinc supplement experiment and lymphocyte proliferation assay

The spleens were removed from induced tumor bearing animals and the splenocytes separated and washed by routine immunological techniques. The splenocytes were counted and plated in a 48 well plate at a concentration of 1 x 10^6^ cells/well. To study the effect of zinc, to each well, 25 *µ*M of zinc chloride was added. For control study, conA (1 *µ*g/ml) and PBS alone were added to the wells and incubated for 36 hours at 37 °C in RPMI 1640 medium supplemented with 10% FCS and 10 mM Hepes in a final volume of 400 *µ*l/well. After 36 hours, the cells were removed, washed and counted in neuber chamber. Six samples were used for each group. The experiment was repeated several times. Results from one representative experiment are given in the result section.

### Statistics

The statistical analysis was done by Student’s t-test.

The work was done at Tumor Immunity and Gene Therapy Unit, Chittaranjan National Cancer Institute, Calcutta-700025, India. The AUP was duly approved by the Animal ethics committee of the Chittaranjan National Cancer Institute, Calcutta, India, a national Institute dedicated to cancer research funded by Government of India.

## Results

### The SZC is depressed under different malignant condition.

**Figure 1:**
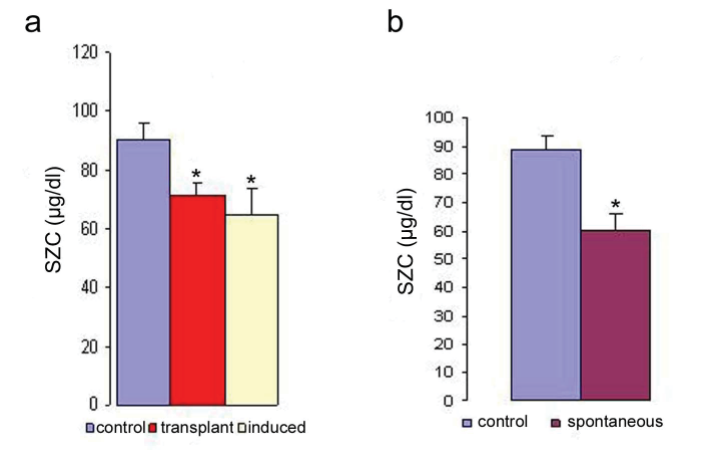
Serum zinc concentration in transplanted, induced (a) and spontaneous (b) tumor bearing mice. The serum zinc concentration was determined in the different types of tumor. The zinc concentration is expressed as µg/dl. Data are shown as mean ± SEM. The results were considered statistically significant when the values in the experimental group were significantly different from controls (* p < 0.05).

The normal level of zinc in the serum (Table 1) was more or less in the same range in the two strains of mice used for this study. The SZC of strain-A mice was slightly higher (90.2 *µ*g/dl) compared to that of C_3_H mice (89.1 *µ*g/dl). However, the evaluation of SZC under different malignant conditions revealed that there was a decrease in the concentration of zinc in all the three types of malignancies studied (spontaneous, induced and transplanted) when compared to their normal counter parts (Fig. 1). The figure 1a shows the SZC in induced and transplanted tumors (mean value of serum zinc on 17th day of transplantation). Figure 1b shows the serum zinc concentration in the spontaneous tumor when compared to controls. It is evident from both the figures that a definitive correlation exists between tumor load and trace element concentration. And the depression of SZC was observed to be maximum in the systemic tumors (mammary carcinoma and fibrosarcoma) compared to the transplanted tumors. The maximum reduction in SZC was in the spontaneous group (33%) followed by that of chemically induced tumor (28%). The minimum reduction in SZC was observed in the animals with transplanted tumors (10% - 18%). The average difference in depression of SZC in the systemic tumors (31.5%) was much higher compared to that of the transplanted group (14.5%). It may be due to greater requirement of zinc in the systemic form rather than in the transplanted tumor which grows as a surrogate tumor in the peritoneal cavity of the host. A detailed study of the SZC in the transplanted tumor bearing animals was done to associate the trace element concentration with the progression of the disease (tumor load). The correlation between the progression of transplanted tumor load and the trace element concentration at different time points is summarized in Table 2. The range of decrease in SZC in the various tumor systems studied varied from 10 to 33 percent when compared to their controls. Among them, the lowest level was noted to be in the DL (10%) followed by S-180 (15%) and EAC (18%) respectively at 17 day of tumor cell transplantation. However, we observed in our earlier studies that the level of serum copper (SCL) increased with the progression of the disease [12].

**Table 1:** Serum Zinc Concentration in Normal Controls

Control	Number of animals	Serum Zinc concentration (µg/dl)
Male strain A	30	90.2 ± 5.1
Female C3H	10	89.1 ± 5.6

The zinc concentration is expressed as µg/dl. The results are shown as mean ± SEM.

**Table 2:** Serum Zinc Concentration is in Transplanted Tumor Bearing Mice at Different Time Points Post Tumor Transplantation

Animal	Number	Day	Cell count	Serum Zn conc. (µg/dl)
Control	15	-	-	90.2 ± 5.6
S-180	45	7	30.9 ± 5.2	83.2 ± 5.0
		10	59.2 ± 7.2	80.1 ± 6.1
		17	196.2 ± 2.1	76.9 ± 3.3*
Ehrlich	45	7	13.8 ± 3.8	82.1 ± 5.0
		10	68.0 ± 3.9	79.0 ± 4.1
		17	230 ± 15.2	74.2 ± 3.1*
Dalton	45	7	45.3 ± 2.6	86.1 ± 5.1
		10	105.2 ± 5.2	84.1 ± 4.2
		17	290.3 ± 10.5	82.3 ± 4.5

The serum zinc concentration was determined in the different transplanted tumor bearing animals after inoculating 1 x 10^7^viable tumor cells in the peritoneal cavity. Forty-five animals were used in each class of transplanted tumors. In all the three types of tumors there was depression in SZC. The zinc concentration is expressed as µg/dl. The results are shown as mean ± SEM. The star (*) indicates statistically significant at p < 0.05.

### The SZC and CZR are increased following tumor regression.

**Figure 2:**
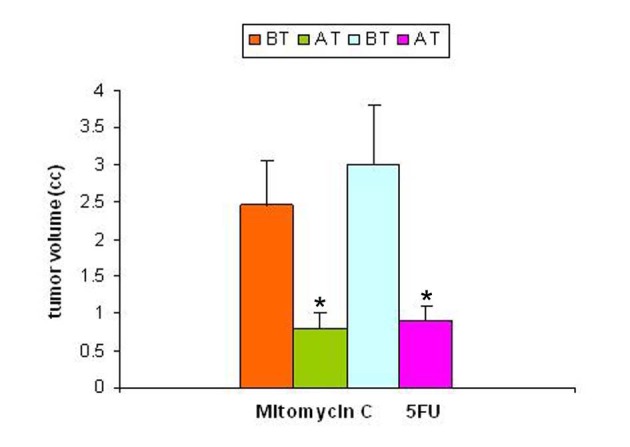
Tumor volume pre- and post-treatment with anti-cancer drugs. The tumor volume was determined prior to and following treatment with two anti-cancer agents 5-flurouracil and mitomycin C. In both the groups there was a significant decrease in tumor volume following treatment (p < 0.05).

Studies carried out with a solid tumor model to evaluate the effect of tumor regression following treatment on SZC showed that the regression of tumor volume following treatment with anti-cancer agents (mitomycin-C and 5-FU) were significant. The results of regression in tumor volume after treatment are shown in Figure 2. The regression in tumor volume was slightly more significant in the mitomycin treated animals (40%) compared to 5-FU (25%) treated animals.

**Figure 3:**
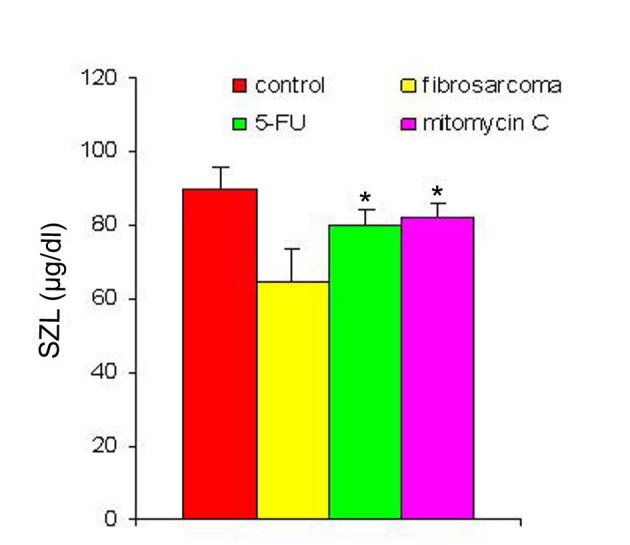
SZL pre- and post-treatment with anti-cancer drugs. The serum zinc concentration was determined prior to and following treatment with two anti-cancer agents. The zinc concentration is expressed as µg/dl. Data are shown as mean ± SEM. In both the groups there was significant elevation in SZL following treatment with anti-cancer agents. * Indicates level of significance p < 0.05.

**Figure 4:**
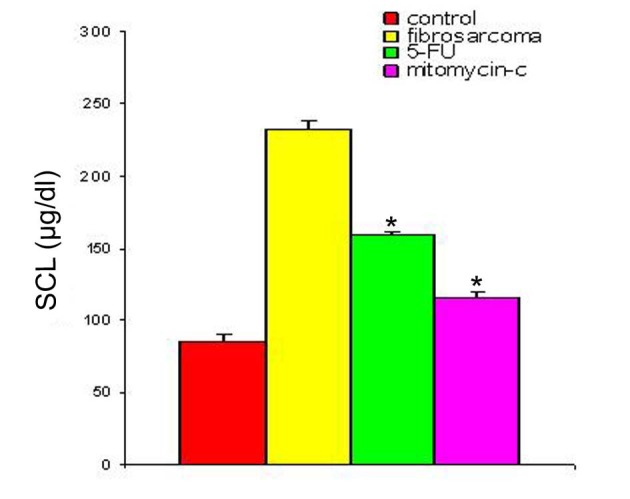
SCL in animals with fibro-sarcoma pre- and post-treatment with anti-cancer drugs. The serum copper concentration was determined prior to and following treatment with two anti-cancer agents. The copper concentration is expressed as µg/dl. Data are shown as mean ± SEM. In both the groups there was significant depression in SCL in the induced tumor bearing animals following treatment with anti-cancer agents. * Indicates level of significance p < 0.05.

**Figure 5:**
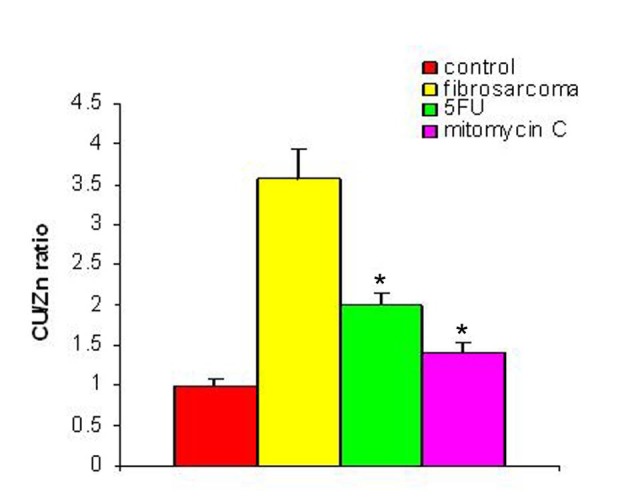
Cu/Zn ratio pre- and post-treatment with anti-cancer drugs. The figure shows the value of copper/zinc ratio prior to and following treatment with two anti-cancer agents in the induced tumor bearing animals. * Indicates level of significance p < 0.05.

With regression in tumor volume following treatment with anti-cancer drugs, the level of SZC was observed to be elevated in both the groups as is shown in Figure 3. The extent of elevation was almost in the same order of magnitude in the two groups of treated animals (∼ 23%) when compared to the untreated group. However, in both the groups, the level of SZC did not reach to the level of controls suggesting the possibility of the presence of a residual tumor burden even after treatment with anti-cancer agents. However, unlike SZC, the SCC that was elevated during malignancy went down significantly following treatment in both the groups as shown in Figure 4. The elevation in SCC was 50% in Mitomycin treated animals compared to 30% in 5-FU treated animals compared to untreated groups. It is difficult to ascertain the plausible reason for this variation between the two groups of treated animals. However, the results do indicate the importance of evaluating SCC as a non-specific marker for the disease activity. In order to ascertain whether copper/zinc ratio (CZR) could be of any importance for assessing the disease activity, the CZR was determined in the two treated groups. The results are shown in Figure 5. It shows that CZR was significantly reduced in both the groups as a result of tumor regression. It is clear from the Figure 5 that the CZR is a better monitor for assessing disease activity than either of them alone. Interestingly, the CZR was a better parameter in assessing the regression in the mitomycin treated animals compared to the 5-Fu treated animals. It was also interesting to note that the state of ‘tumor waning’ was maintained for a considerable period of time even long after tumor regression suggesting that there was additional control mechanism operative in the host. It could be that the appropriate immunity against the tumor was operative following tumor regression as a result of elevation of zinc concentration. It was our hypothesis that zinc could be playing an important role in restoring tumor immunity following tumor regression which could impact tumor progression. In order to ascertain the conjecture, we carried out a supplementation experiment using splenocyte from tumor bearing host.

### Supplementation of zinc increases proliferation of splenocytes of tumor bearing animals.

**Figure 6:**
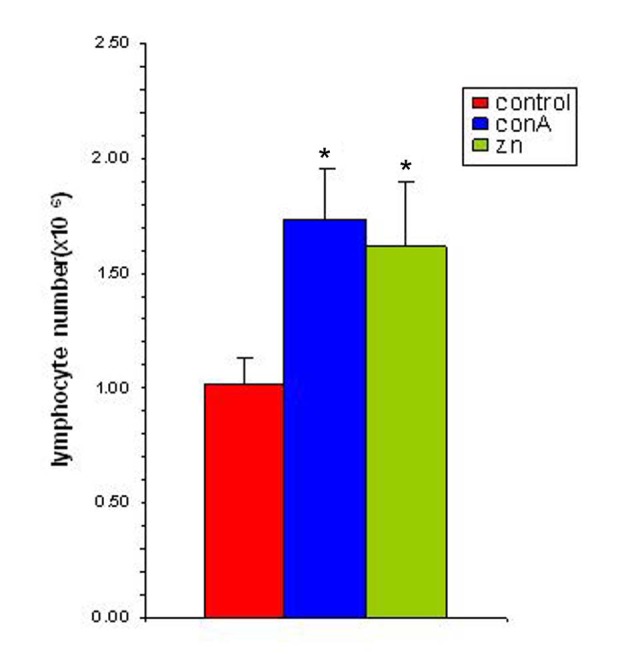
Zinc supplementation increases splenocyte proliferation in vitro. The splenocyte proliferation was determined following supplementation with zinc (25 µM) and in presence of ConA (1 µg/ml). The results are expressed as mean ± SEM. * Indicates level of significance p < 0.05.

Studies with zinc supplementation revealed that there was a significant increase in the splenocyte number following incubating the splenocytes of tumor bearing animals with Zn (Figure 6). The splenocytes from tumor bearing animals when exposed to 25 *µ*M of zinc showed a significant amplification and this increase was comparable to that of conA (1 *µ*g/ml) stimulated spleen. This suggested that the splenocytes of tumor bearing animals were competent to respond to an antigenic stimulus while in presence of zinc.

## Discussion

The present study dealing with the importance of zinc, SZC and to some extent SCC during malignancy and following successful treatment reveals that there was a change in the level of SZC with induction of tumor and it inversely correlated with the progression of a tumor. Depression of SZC has also been observed in several human malignancies [13]. In this study, SZC showed an inverse correlation with the progression of the disease. It was significantly reduced in both the spontaneous and induced forms of malignancy. This is of significance because all known human malignancies, which fall in the two broad categories, also show a similar trend in respect of the level of SZC, pointing that a common mechanism may be operative in both human and animal systems. Extensive studies done with the solid form of induced tumor established the importance of estimating the concentration of Zn, in addition to that of copper, in assessing the disease activity. In the systemic form of the disease, with regression of the tumor mass, there was a significant elevation in the SZC and depression in the SCC compared to their prior levels respectively. This was also reflected on the value of CZR. With the onset of tumor regression, a significant reduction in CZR was noted in the tumor bearing animals compared to their untreated levels. Since the state of ‘regression’ in the tumor mass was maintained for a considerable period of time following treatment with anti-cancer agents, it is likely that other mechanism(s) could be operative in controlling the tumor growth. Therefore, it was hypothesized that the elevated zinc levels following tumor regression could be playing an indirect role in controlling the extent of tumor growth, by influencing the immune response of the host.

A correlation between the concentration of different trace elements and the malignant state have been reported earlier for different forms of human [14, 15] and animal malignancies [16]. We also reported similar correlation earlier, with respect to copper in many different forms of tumor in humans and in experimental tumor systems [10, 12]. In the present study, we have extended our previous observation that a direct correlation exists between trace element concentration and malignancy and established that same may be true with respect to depressed Zinc concentration in different forms of tumors. Similar lower levels of Zinc concentration have been reported recently in cancer of the digestive tract [17], and in different forms of leukemia [3, 18]. The decrease in SZC has been suggested to serve as an index for unfavorable progression of acute leukemia [3]. A decrease in zinc-alpha2-glycoprotein (AZGP1) mRNA levels in malignant prostate epithelium has been shown to predict biochemical recurrence, as defined by rising levels of serum PSA after radical prostatectomy. The authors suggested that the measurement of AZGP1 levels in radical prostatectomy specimens could permit an accurate and timely assessment of risk of metastatic progression after radical prostatectomy [19]. Similarly, it was considered that determination of zinc levels could also be used as a diagnostic or screening tool and could lead to the formulation of techniques to utilize zinc to evaluate prostatic pathology [20]. In our study also it was observed that the SZC was a good prognostic tool following treatment with anti-cancer drugs. However, given the variation in the SCC in the two groups of treated animals, the estimation of CZR appears to be a better monitor for evaluating the prognosis. But it is prudent to point out that such parameters are somehow influenced by the type of anti-cancer agent used for the treatment. It is also interesting to note that the state of ‘tumor waning’ was maintained for a considerable period of time even long after tumor regression suggesting that there was additional control mechanism operative in the host. It could be that appropriate immunity against the tumor was operative following tumor regression as a result of elevation of zinc concentration. It is our hypothesis that zinc could play an important role in restoring tumor immunity following tumor regression.

Recent insights in trace element research are beginning to demonstrate the relevance of zinc in immune related activities. It has been observed that zinc can induce macrophages to produce increased amounts of macrophage migration inhibitory factor (MIF) [21], which is a pro-inflammatory cytokine whose expression is critical to generation of antigen-specific immune response. Role of zinc in T cell development was established from studies made during zinc deficiency. Reports illustrated that there was a reduction in the proportion of TCRαβ+ cells that express CD90 (Thy 1.1), a marker found on recent thymic immigrants that took part in thymocyte development and post thymic maturation, in the blood and spleen of zinc deficient rats [22-24]. With repletion in zinc levels, the number of TCRαβ+ cells was raised to normal levels within seven days. Thus zinc seems to play a direct role in recruiting and maintaining T cells in the blood. Not only that, studies also showed that Zn is required for maturation of T cells from CD4^-^CD8^+^ condition to CD4^+^CD8^+^ state. This was substantiated by the finding from adult Zn deficient mice, wherein there was a substantial reduction in percentage (38%) of thymic CD4^+^CD8^+^ cells and a greater proportion of CD4^-^CD8^-^, CD4^+^CD8^-^, and CD4^-^CD8^+^ T cells [25]. Zn could also be participating in maintaining the cytotoxic T lymphocyte (CTL) pool through manipulation of p56^lck^ protein which is expressed on T cells. Higher levels of p56^lck^ protein observed to be expressed on T cells during zinc deficiency could trigger apoptosis in T cells as p56^lck^ is known to signal for the initiation of apoptosis [26, 27]. Thus lower zinc concentration could be responsible for the destruction of T cells by triggering apoptosis and impairing immune response. During malignancy, a tumor present in a constant zinc deficient environment could promote unrestricted growth of the tumor as there would be a steady depletion of antigen specific T cells from the blood. This may possibly trigger an early onset of metastasis in tumor bearing host. This contention was further strengthened by the report that with Zinc restriction, in humans, there was a tendency for a lower ratio of CD4+CD45RA+ (naive) compared to CD4+CD45RO+ (memory cells) that are vital for initiating secondary immune response. Our finding that zinc could aid in the normal proliferation of splenocytes of tumor bearing animals further supports this proposition. Therefore, supplementing with zinc during malignancy may help reverse the tumor environment from ‘zinc deficient state’ to ‘zinc sufficient state’ that could be of significance in stimulating T cell immigration to the tumor vicinity as well as increasing the chances for availability of antigen presenting cells at the tumor vicinity. The environment could be congenial not only in increasing the number of T cells that become antigen specific CTL, but also persuade in maintaining a pool of memory T cells (T_m_) in the host, which could impact on curbing metastasis. Thus zinc supplementation could have a far reaching effect in controlling metastasis or slowing down the growth of existing tumor which could provide vital time for appropriate therapeutic intervention.

Overall, our present study confirms that zinc concentration is decreased during malignancy which may be relevant in immuno-pathology of the tumor. The estimation of SZC is of significance in assessing the extent of the disease. Determination of zinc levels as a diagnostic marker or as a screening tool during malignancy has also been recently suggested in humans by others [21]. Also, zinc influences lymphocyte proliferation that could directly impact on the immune response of tumor bearing host.

From our studies, we propose the use of CZR for assessing the disease activity and specifically for monitoring tumor regression. We also hypothesize that supplementing zinc to malignant subjects prior to and following therapeutic intervention with anti-cancer agents could help improve their immune response against tumor and thereby regulate tumor growth and delay the onset of metastasis.
